# Development of an Ultrasensitive Immunoassay for Detecting Tartrazine

**DOI:** 10.3390/s130708155

**Published:** 2013-06-25

**Authors:** Zhuokun Li, Shanshan Song, Liguang Xu, Hua Kuang, Shidong Guo, Chuanlai Xu

**Affiliations:** State Key Laboratory of Food Science and Technology, School of Food Science and Technology, Jiangnan University, Lihu Road 1800, Wuxi 214122, China; E-Mails: lzk669@126.com (Z.L.); sss@jiangnan.edu.cn (S.S.); xuliguang2006@126.com (L.X.); khecho@163.com (H.K.); guosd@jiangnan.edu.cn (S.G.)

**Keywords:** tartrazine, ELISA, monoclonal antibody, beverage

## Abstract

We have developed an ultrasensitive indirect competitive enzyme-linked immunosorbent assay for the determination of tartrazine. Two carboxylated analogues of tartrazine with different spacer lengths, and one derivative from commercial tartrazine after a little chemical modification, were synthesized as haptens in order to produce antibodies specific to tartrazine. The effect of sulfonic acid groups on the hapten structure of tartrazine was also studied carefully for the first time. A most specific monoclonal antibody against tartrazine was created and exhibited an IC_50_ value of 0.105 ng/mL and a limit of detection of 0.014 ng/mL, with no cross-reactivity to other structurally-related pigments. The established immunoassay was applied to the determination of tartrazine in fortified samples of orange juice and in real positive samples of carbonated beverages.

## 1. Introduction

Tartrazine (trisodium 5-hydroxy-1-(4-sulfonatophenyl)-4-(4-sulfonatophenylazo)-H-pyrazol-3-carboxylate) is also known as FD&C Yellow no. 5 and E 102. It is an artificially synthesized azo pigment and its use is permitted as a colorant in food products, cosmetics and pharmaceuticals, with a recommended acceptable daily intake (ADI) of 7.5 mg/kgbw. However long-term and excessive ingestion of tartrazine may cause a variety of adverse effects [1–9]. Mpountoukas *et al.* indicated that tartrazine had genotoxic potential towards human lymphocytes and could bind directly to DNA [10]. Kashanian *et al.* also reported similar results and pointed out that tartrazine was potentially toxic to calf thymus DNA *in vitro* [4]. A study by Tanaka *et al.* reported that tartrazine could exert adverse effects on neurobehavioral parameters [8], while Gao *et al.* indicated that tartrazine could cause neurotoxicity and deficits in learning and memory in mice and rats [11]. Li and co-workers investigated the toxic interaction between tartrazine and bovine hemoglobin (BHb), and found that tartrazine had an obvious toxic effect [12]. Due to this potential toxicity, it is crucial to control the amount of tartrazine used in food products and it is therefore necessary to develop analytical methods capable of evaluating the exposure of the general population to tartrazine.

To date, various methods have been reported for the detection of tartrazine, such as chromatography [13–15], spectrophotometry [16–18], electroanalytical methods [19–21], and novel nanosensor detection methods [22,23]. However, most of these methods are expensive, time consuming or complicated, and therefore not suitable for routine extensive monitoring of tartrazine. In contrast, an enzyme-linked immunosorbent assay (ELISA) could be an ideal alternative technology, due to its high sensitivity, time-efficiency and cost-effectiveness. To date, there have been several reports of development of an immunoassay for tartrazine [24,25]. However, to the best of our knowledge, the reported most sensitive antibody against tartrazine was produced for the determination of tartrazine in human urinary samples and could only exhibit an IC_50_ value of 7.7 ng/mL [25]. In this study, we developed an ultrasensitive immunoassay based on a specific monoclonal antibody to detect tartrazine in beverages.

## 2. Materials and Methods

### 2.1. Reagents

Pigment standard substances (erythrosine, tartrazine, crystal violet, malachite green, sunset yellow, new coccine, allura Red AC, orangeim, auramine O, sudan II, solvent red 24, solvent red 1, indigotin and chrysodine) were purchased from Sigma Chemical Co. (St. Louis, MO, USA). Carrier proteins such as keyhole limpet hemocyanin (KLH), bovine serum albumin (BSA) and ovalbumin (OVA) were also obtained from Sigma, as were coupling agents including isobutyl chloroformate, tri-*n*-butylamine, *N*-hydroxysuccinimide (NHS) and 1-(3-dimethylaminopropyl)-3-ethyl carbodiimide (EDC), as well as polyethylene glycol solution (PEG, Hyb-ri-Max, 50% (w/v)) as the fusogen. Goat anti-mouse IgG conjugated to horseradish peroxidase (HRP) and 3,3,5,5-tetramethylbenzidine (TMB) were bought from Sino-American Biotechnology Co. (Luoyang, China). Freund's complete and incomplete adjuvant and dimethyl sulfoxide (DMSO) were purchased from Sigma. RPMI-1640 cell culture medium and fetal calf serum (FCS) were obtained from Sunshine Biotechnology Co. (Nanjing, China). Hypo-xanthine-aminopterin-thymidine medium (HAT), and hypoxanthine-thymidine medium (HT) were bought from Gibco BRL Co. (Paisley, UK). All other chemicals and reagents of analytical grade were obtained from Sinopharm Chemical Reagent Co, Ltd. (Shanghai, China).

### 2.2. Instrumentation

An Avance III 400 MHz Digital NMR Spectrometer (Bruker Biospin, Billerica, MA, USA) was used for hapten characterization. A Biomate 3S UV-Visible Spectrophotometer (Thermo Fisher Labsystems, Waltham, MA, USA) was used for ultraviolet-visible absorption measurements. All 96-well, 24-well and 6-well cell culture plates, as well as 96-well polystyrene microplates were obtained from Costar (Costar Inc., Milpitas, CA, USA). Immunoassay absorbance was measured at a wavelength of 450 nm using the Multiskan MKS Microplate Reader (Thermo Fisher Labsystems). Hybridoma cells were cultured in CO_2_ Incubators (Thermo Fisher Labsystems).

### 2.3. Buffers

In this study, PBS buffer was prepared with 0.01 M phosphate buffered saline, 144.3 mM sodium chloride and 2.68 mM potassium chloride, and the pH of the final solution was adjusted to 7.4. The coating buffer was 0.05 M carbonate buffer (CB, pH 9.6), and blocking buffer was prepared from coating buffer with the addition of 0.2% casein. Substrate solution was prepared in 0.048 M citric acid, 0.103 M disodium hydrogen orthophosphate (Na_2_HPO_4_·12H_2_O), 0.18% (v:v) hydrogen peroxide (H_2_O_2_, 30%) and 2.496 mM 3,3′,5,5′-tetramethylbenzidine (TMB). Stop solution was 2M sulfuric acid (10%, v/v).

### 2.4. Hapten Synthesis

**Scheme 1.**
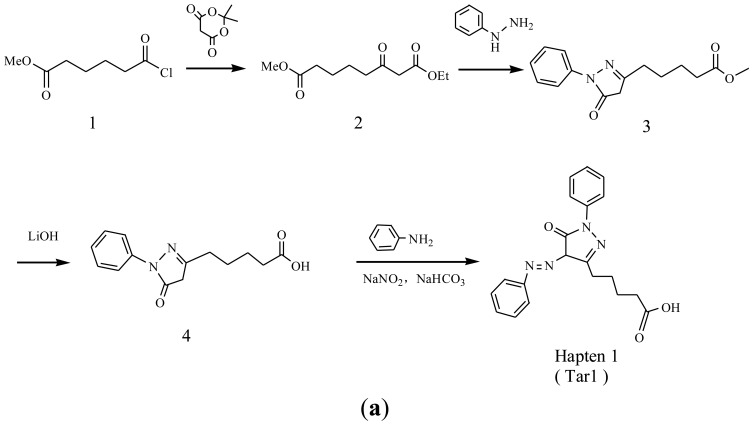

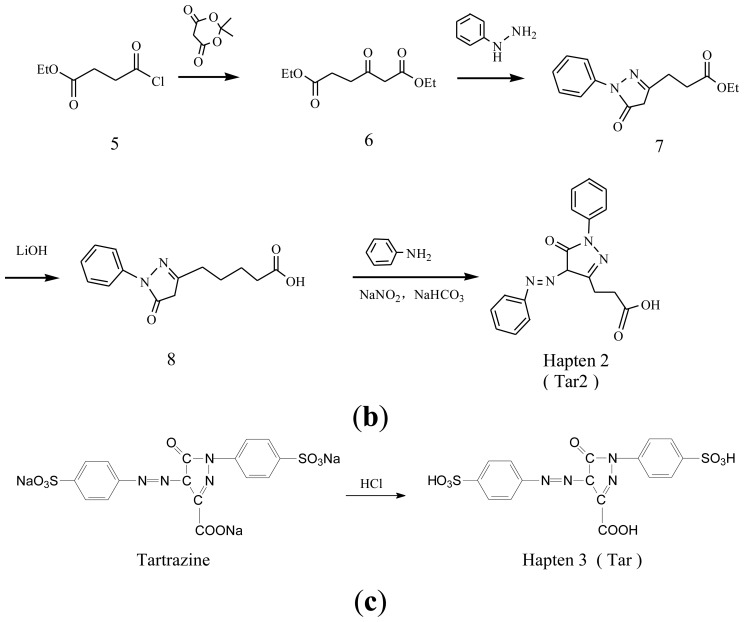
(**a**) Synthesis of tartrazine hapten 1 (Tar1); (**b**) Synthesis of tartrazine hapten 2 (Tar2); (**c**) Synthesis of tartrazine hapten 3 (Tar).

Three haptens of tartrazine used for immunizing and coating antigens were employed in this experiment, and their structures and synthesis routes are shown in Scheme 1. Hapten 1 and hapten 2 were synthesized by the same procedure using corresponding initial raw chemicals. The detailed synthesis procedure and the characterization of haptens are described in the following subsections.

#### 2.4.1. 1-Phenyl-3-valeric acid-4-phenylazo-5-pyrazolone azo (Hapten 1)

2,2-Dimethyl-1,2-dioxane-4,6-dione (6.674 g, 25.49 mmol) was stirred with dichloromethane (10 mL) and pyridine (4.077 g, 51.54 mmol) until the solid dissolved. A solution of methyl 6-chloro-6-oxohexanoate (4.95 g, 27.71 mmol) in dichloromethane (5 mL) was added dropwise to the stirring solution of Meldrum's acid at 0–3 °C under nitrogen over a period of 1 h and then the mixture was stirred for a further 1 h. The reaction product was washed with hydrochloric acid (HCl, 6N) and water, dried over anhydrous Na_2_SO_4_, and concentrated *in vacuo*. The residue was stirred and refluxed with absolute ethanol (50 mL) for 1 h. Following evaporation of the solvent, methyl *tert*-butyl ether (MTBE, 50 mL) was added to dissolve the residue, and the residue was washed with water (3×). Finally, the extracted product was dried and concentrated to give compound **2** (5.32 g, 83%). ^1^H-NMR (CDCl_3_): δ 4.25–4.05 (m, 2H), 3.64 (s, 3H), 3.41 (s, 2H), 2.63–2.45 (m, 2H), 2.40–2.20 (m, 2H), 1.72–1.53 (m, 4H), 1.30–1.20 (m, 3H).

Phenylhydrazine hydrochloride (3.62 g, 25 mmol) was added portionwise to a stirred solution of compound **2** (4.8 g, 20.84 mmol) in absolute ethanol (200 mL) at room temperature, then the reaction mixture was stirred at room temperature for 16 h. The solvent was evaporated under reduced pressure, and the product was purified by chromatography on silica gel with petroleum ether and ethyl acetate (PE:EA = 1:1 to 1:3) to give compound **3** (3 g, 52%). ^1^H-NMR (CD_3_OD): 7.62 (d, J = 7.6 Hz, 2H), 7.49–7.45 (m, 2H), 7.35–7.29 (m, 1H), 3.71–3.58 (m, 3H), 3.34–3.25 (m, 2H), 2.65–2.50 (m, 2H), 2.45–2.31 (m, 2H), 1.80–1.61 (m, 4H).

Lithium hydroxide (LiOH, 1.147 g, 27.34 mmol) dissolved in water (10 mL) was added to a stirred solution of compound **3** (3 g, 10.9 mmol) in ethanol (25 mL) and THF (25 mL) at room temperature, and the mixture was stirred overnight. The solution was washed with ethyl acetate (EA), then adjusted to pH 4–5 using hydrochloric acid (HCl, 1N). Following extraction by ethyl acetate, the extracted product was dried and concentrated to give compound **4** (2 g, 70%). ^1^H-NMR (DMSO-*d*_6_): δ 12.01 (s, 1H), 11.45 (s, 1H), 7.71 (d, *J* = 7.6 Hz, 2H), 7.49–7.37 (m, 2H), 7.30–7.15 (m, 1H), 5.39 (s, 1H), 2.50–2.40 (m, 2H), 2.30–2.15 (m, 2H), 1.70–1.46 (m, 4H).

A solution of aniline (2 g, 21.475 mmol) in hydrochloric acid (concentrated HCl/H_2_O = 8.95 mL/20 mL) was cooled to 5 °C in an ice bath, then a cool solution of sodium nitrite (1.778 g, 25.77 mmol) in water (5 mL) was added. The solution was stirred for 1 h to generate the diazo salt. Sodium bicarbonate (NaHCO_3_, 387 mg, 4.61 mmol) and sodium carbonate (Na_2_CO_3_, 4 g, 37.73 mmol) were added to a stirred solution of compound **4** (0.8 g, 3.073 mmol) in methanol (MeOH, 1 mL) and water. The solution was stirred until compound 4 dissolved, and the diazo salt solution was then added at 0–10 °C over 1 h, and stirred for another 4 h. Then, the solution was adjusted to pH 5 using concentrated hydrochloric acid. The reaction mixture was extracted with ethyl acetate, dried and concentrated, and finally purified by pre-HPLC to give hapten 1 (0.6 g, 53%). ^1^H-NMR (DMSO-*d*_6_): δ 13.31 (s, 1H), 12.04 (s, 1H), 7.93 (d, *J* = 8.0 Hz, 2H), 7.62 (d, *J* = 8.0 Hz, 2H), 7.52–7.42 (m, 4H), 7.24–7.23 (m, 2H), 2.80–2.68 (m, 2H), 2.36–2.23 (m, 2H), 1.84–1.77 (m, 2H), 1.70–1.61 (m, 2H). UPLC-TOF-MS/MS [negative mode] *m*/*z*: 363 [M-H]^−^.

#### 2.4.2. 1-Phenyl-3-propionic acid-4-phenylazo-5-pyrazolone azo (Hapten 2)

Hapten 2 was synthesized by the same synthesis procedure used for hapten 1, described above (Scheme 1) using ethyl 4-chloro-4-oxobutanoate as the starting material. The final extraction of 1-phenyl-3-propionic acid-4-phenylazo-5-pyrazolone azo (0.7 g, 32%) was also characterized by nuclear magnetic resonance. ^1^H-NMR (DMSO-*d*_6_): δ 13.29 (s, 1H), 12.28 (s, 1H), 7.93 (d, *J* = 8.0 Hz, 2H), 7.64 (d, *J* = 8.0 Hz, 2H), 7.52–7.40 (m, 4H), 7.30–7.19 (m, 2H), 3.00–2.90 (m, 2H), 2.79–2.72 (m, 2H). UPLC-TOF-MS/MS [negative mode] *m*/*z*: 335 [M-H]^–^.

#### 2.4.3. 3-Carboxy-5-oxo-1-p-sulfophenyl-4-*p*-sulfophenylazo pyrazole (Hapten 3)

Hapten 3 was generated from original commercial tartrazine after a little modification. Initial concentrated hydrochloric acid was chosen to remove the three sodium from a commercial tartrazine molecule. Briefly, tartrazine (4 g, 7.48 mmol) was added to a 50 mL centrifuge tube, and then concentrated hydrochloric acid (12 M, 40 mL) was poured into the tube while vortexing to dissolve the tartrazine. After 24 h standing in the dark, the tartrazine solution was centrifuged. The supernatant was removed, and the sediment of tartrazine was purified another two times using the same procedure. Finally, hapten 3 was obtained after drying the purified sediment of tartrazine.

### 2.5. Preparation of Immunogen and Coating Antigen

Hapten 1 and hapten 2 were conjugated to keyhole limpet hemocyanin (KLH; for use as the immunogen) or ovalbumin (OVA; for use as the coating antigen) by the active ester method. Briefly, to a stirred solution of each hapten (0.05 mmol) in N,N-dimethylformamide (0.8 mL), N-hydroxysuccinimide (NHS, 0.1 mmol) was added at room temperature, and 1-(3-dimethylaminopropyl)-3-ethyl carbodiimide (EDC, 0.1 mmol) was also added 15 min later. This activation reaction was carried out for 2 h to give solution A. Solution A was then centrifuged (8,000 × g, 8 min), and the supernatant was acquired and added dropwise to a stirred solution of carrier protein (0.02 μmol KLH or 1.25 μmol OVA dissolved in 10 mL PBS). The reaction mixture was stirred overnight at ambient temperature to complete the coupling reaction. The immunogenic or coating antigen mixture was then dialyzed against PBS for 4 days and characterized using an ultraviolet spectrophotometer, and finally stored at −20 °C until use.

The mixed anhydride reaction was employed to conjugate hapten 3 with carrier protein. The carboxyl group of hapten 3 was covalently coupled to the amino group of KLH (for use as immunogen) or OVA (for use as coating antigen). A stirred solution of hapten 3 (0.02 mmol) dissolved in dimethyl sulfoxide (DMSO, 0.5 mL) and N,N-dimethylformamide (DMF, 0.1 mL) was cooled to 0 °C in an ice bath, and then tri-*n*-butylamine (0.06 mmol) was added. The solution mixture was reacted at 0 °C for 0.5 h with continuous stirring. Next, isobutyl chloroformate (0.06 mmol) was added, and reacted for another 2h in an ice bath. After centrifugation of the reaction mixture, the supernatant was dropped very slowly into a cold protein solution (0.01 μmol KLH or 0.5 μmol OVA dissolved in 5 mL PBS). The conjugation reaction continued for 12 h at 4 °C, and was then terminated by dialysis to give an immunogen or coating antigen.

### 2.6. Pab and Mab Production and Characterization

An immunization program was carried out with minor modification of a reported format [26–28]. Each immunogen was emulsified with Freund's adjuvant using an emulsifier to immunize six female BALB/c mice (6–8 weeks old). The first immunization was carried out by subcutaneous injection at a dose of 80 μg immunogen per mouse, using Freund's complete adjuvant for emulsification in advance. The booster inoculations were implemented every three weeks with the immunogen (50 μg per mouse) emulsified with Freund's incomplete adjuvant to meet the requirements of serum titer. The inhibition of serum from tail blood was monitored by icELISA. The sprint immunization was administered by intraperitoneal injection three days before the mouse was sacrificed for cell fusion. The ocular blood was also collected to provide serum to obtain a polyclonal antibody.

The spleen from an immunized mouse (M2) with the highest specificity of the tartrazine antibody was separated aseptically and the splenocytes were fused with Sp2/0 myeloma cells by adding 50% PEG 1500 over 1 min. The cell fusion was terminated by adding cell culture solution, and the hybridoma cells produced were transferred to 96-well plates and cultured in a selective medium consisting of RPMI 1640 medium, 20% FCS and 2% HAT solution. After incubating for 9 days, the cell supernatants were assayed by icELISA for cell selection. The selected hybridomas were subcloned four times using the limiting dilution method [29]. The cell lines chosen were injected intraperitoneally into mice to secrete monoclonal antibodies. Ascites was extracted from the mice and stored at -20 °C after purification with caprylic acid and saturated ammonium sulfate [30].

### 2.7. Indirect Competitive ELISA (icELISA)

The procedure of this indirect competitive ELISA was similar to a previously reported description [29]. The titer of the antibody and the optimal combination of coating antigen and antibody were determined by checkerboard titration [28] to achieve the best sensitivity. The hapten-OVA diluted in carbonate buffer was used to coat 96-well polystyrene microplates (100 μL/well) for 2 h at 37 °C which were then blocked with blocking buffer for another 2 h. Serial dilution of tartrazine analyte in PBS (50 μL/well) and antibody diluents (50 μL/well) were distributed to the microplates and incubated for 30 min at 37 °C. Then, goat anti-mouse IgG-HRP (100 μL/well) was added and plates were incubated for another 30 min. The enzyme reaction was triggered by adding TMB substrate solution (100 μL/well) and terminated by adding sulfuric acid (2M, 50 μL/well), and the corresponding absorbance was read by a microplate reader at 450 nm. Competitive inhibition curves were generated by plotting inhibition (B/B_0_) against the logarithmic concentration of tartrazine standard. Sigmoid curves were simulated by the four-parameter logistic equation using Origin 8.5 software.

### 2.8. Cross-Reactivity

The sensitivity and specificity of the tartrazine monoclonal antibody secreted by hybridoma cells were characterized by the half-maximal inhibitory concentration (IC_50_) and cross-reactivity (CR). Thirteen pigments with related structures were selected to test cross-reactivities (Table 1). The CR values were calculated as follows: CR(%) = [IC_50_ (tartrazine)/IC_50_(interferent)] × 100%.

### 2.9. Sample Preparation and Detection

A well-known brand of orange juice was obtained from a local supermarket and was chosen to carry out a recovery test for tartrazine. The listed ingredients on the label of the orange juice stated that it contained water, sugar, citric acid, malic acid, sodium citrate, ascorbic acid, arabic gum, 10% juice and natural pulp. The label did not include tartrazine additive and the orange juice was confirmed to be negative by UPLC-TOF-MS/MS. The acceptable maximal addition of tartrazine in beverage permitted by Chinese law is 0.1g/kg of food as consumed. However, considering the existence of tartrazine in real positive beverage samples, three fortified levels (0.5, 1, 2 mg/mL) of tartrazine were created for the recovery experiments. The spiked samples were directly diluted 5,000-fold with PBS for measurement by icELISA, without any filtration or SPE cleanup. Each spiked sample was tested for five replicates. The recoveries were calculated based on the standard curve and the measured values of the fortified orange juice samples. Intra-assay variation was surveyed based on 5 replicates and the coefficient of inter-assay variation was tested on three consecutive days.

Another well-known brand of carbonated beverage which included tartrazine additive in the list of ingredients was selected as the real positive sample to test the developed immunoassay. The carbonated beverage was also diluted appropriately with PBS and directly assayed using the optimized icELISA. Accuracy was evaluated based on the standard deviation and the coefficient of variation.

## 3. Results and Discussion

### 3.1. Synthesis of Tartrazine Haptens

Hapten structure played a key role in the production of highly sensitive antibodies. Both the conjugation position at the hapten molecule and the length of the spacer may influence the production and the sensitivity of a desired antibody. In the present study, three tartrazine haptens were designed and used for further immune response (Scheme 1). Two carboxylated analogues of tartrazine without sodium sulfonates were synthesized by a four-step reaction. Both the derivative with a five carbon-atom spacer length (hapten 1 or Tar1) and the other derivative with a three-carbon-atom spacer length (hapten 2 or Tar2) were all modified at the carboxyl group. These two haptens were characterized by nuclear magnetic resonance (NMR) and mass spectrometry (MS).

The third hapten (Hapten 3 or Tar) was derived from commercial tartrazine after a small chemical modification. The sodium was removed from the tartrazine molecule with concentrated hydrochloric acid (Scheme 1). It was very difficult to separate the sodium and the sulfoacid by cation exchange resin, as was proven by our previous work. We also found that the concentration of hydrochloric acid and the standing reaction time play a crucial role in the sodium removal. Hapten 3 could be easily dissolved in DMSO, while it was difficult to dissolve in DMF, and the water solubility of hapten 3 was considered to be negligible. In contrast, commercial tartrazine was obviously totally water soluble which could be ascribed to the presence of sodium.

### 3.2. Synthesis of Immunogen and Coating Antigen

**Figure 1.**
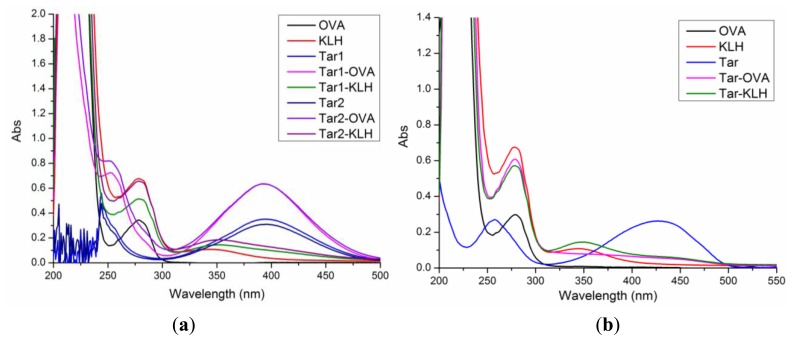
(**a**) UV absorption spectra of hapten 1 and hapten 2 conjugates; (**b**) UV absorption spectra of hapten 3 conjugates.

Two tartrazine derivatives bearing a carboxyl group at the end of the spacer were activated by the active ester method and covalently attached to a carrier protein (KLH or OVA). Their UV absorption spectra are shown in Figure 1(a). Hapten 1 and hapten 2 showed almost the same specific UV absorption peaks at 395 nm and 244 nm. The absorption spectra of Tar1-OVA and Tar2-OVA had the characteristic UV peaks of its hapten at 395 nm and showed superposition properties to give a new peak around 252 nm. All the results shown in Figure 1(a) indicate that hapten 1 and hapten 2 were successfully conjugated to carrier proteins.

Hapten 3 (Tar) was coupled to a protein by the mixed anhydride method. The UV spectra of hapten 3 and hapten 3-protein conjugates are shown in Figure 1(b). It is obvious that hapten 3 had two characteristic UV peaks at 425 nm and 258 nm. The conjugates of Tar-OVA and Tar-KLH were also proven to be successfully synthesized.

### 3.3. Screening of Antibodies

All the hapten-KLH conjugates were used as immunogens, while the conjugates of hapten-OVA were used as coating antigens. The conjugates of Tar1-KLH and Tar2-KLH were primarily emulsified and used to immunize six female BALB/c mice, respectively. The antisera obtained from all these mice after several immunizations were monitored by icELISA and showed no specificity to the tartrazine analyte, whether using Tar1-OVA or Tar2-OVA as the coating antigen. These results indicate that two sodium sulfonate groups in the tartrazine molecule structure might play a crucial role in inducing production of an antibody against tartrazine. They may be a part of the epitope which the antibody recognizes. We concluded that the sulfonic acid groups had strong electron-withdrawing ability, and the absence of the sodium sulfonates could cause the charge distribution of hapten derivatives to significantly differ from that of the tartrazine molecule. However, the charge distribution is crucial for recognition of the antigens by the major histocompatibility complex (MHC), and this has a major influence on the production of antibodies [26,27]. This demonstrates that the sulfonic acid groups of tartrazine play an irreplaceable role in the induction of specific antibody generation, and should be retained in the process of hapten design and synthesis.

**Figure 2.**
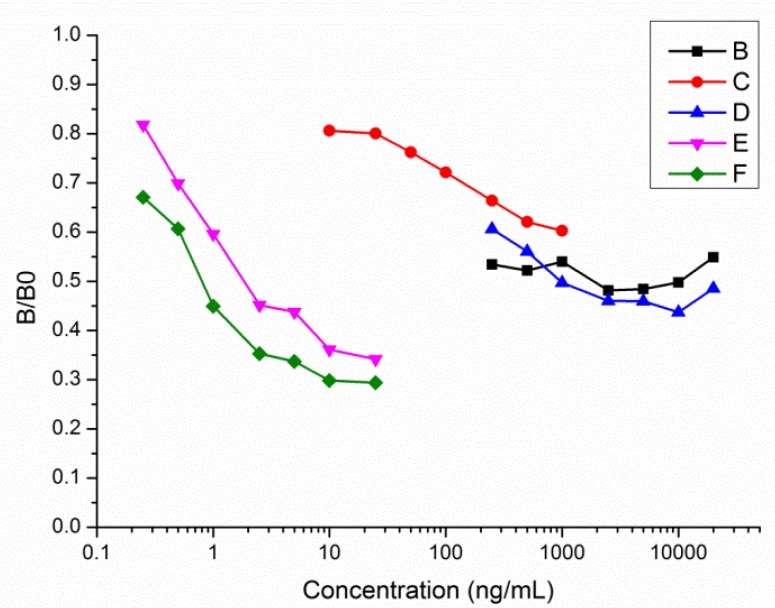
Inhibition curves of polyclonal antibodies from mouse M2 and M3 by icELISA. B represents antiserum from M3 after the fourth immunization; C represents antiserum from M3 after the sprint immunization; D represents antiserum from M2 after the fourth immunization; E represents antiserum from M2 after the fifth immunization; F represents antiserum from M2 after the sprint immunization.

The immunogen Tar-KLH, which was conjugated from Hapten 3, was also used to immunize six female mice. Hapten 3 was modified from commercial tartrazine and contained all the antigenic determinants of tartrazine. The antisera collected from the mice after each booster immunization with Tar-KLH were assayed by icELISA using Tar-OVA as the coating antigen. Two mice (M2 and M3) were found to exhibit better performance in antibody production after immunization. The inhibition curves of the obtained polyclonal antibodies are shown in Figure 2. The sensitivities of the antisera were enhanced substantially with the increasing number of immunizations. However it was obvious that all the inhibition curves were not sensitive to tartrazine when it came to the corresponding high concentration of tartrazine analyte. Apparently, the antisera contained some antibodies which could recognize the coating antigen but could not identify tartrazine. These results further confirmed that the sulfonic groups within the tartrazine structure are a key part of the epitope and could not be modified or reacted. The sulfonic group could be activated to covalently couple with the amino group of the carrier proteins by the active ester method or the mixed-anhydride method. In our previous work, commercial tartrazine was used directly as the hapten to conjugate with the carrier protein, but the antisera obtained did not exhibit any recognition of tartrazine, due to the much higher reactivity of the sodium sulfonate group than the carboxyl group. Although the removal of sodium from the sodium sulfonate group could greatly reduce its ability to be activated, hapten 3 would still be able to conjugate to the carrier protein via the sulfonic group rather than the carboxyl group. In order to solve the problem described above, the hybridoma technology must be employed to produce tartrazine monoclonal antibodies.

### 3.4. Sensitivity and Specificity of the Monoclonal Antibody

**Figure 3.**
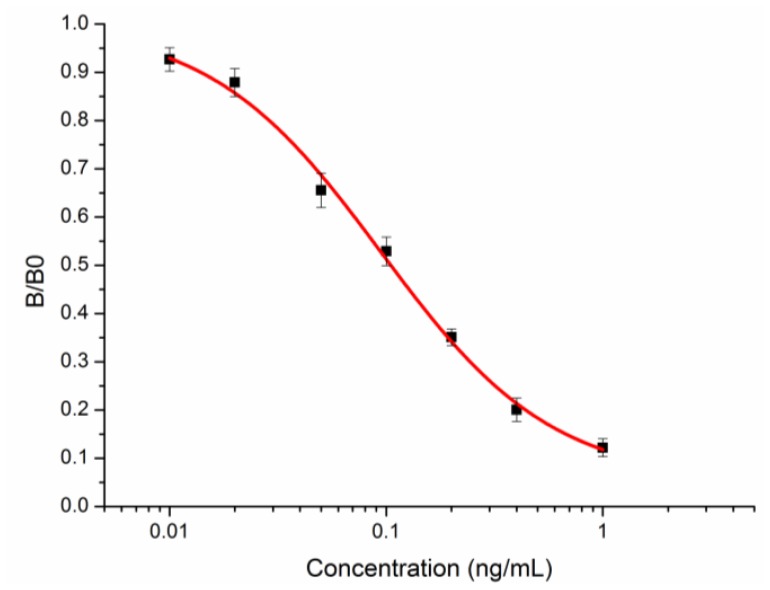
Standard inhibition curve of mAb 1F3 by icELISA.

The mouse M2 injected with Tar-KLH was sacrificed for cell fusion, and a monoclonal antibody 1F3 (mAb 1F3) specific to tartrazine was successfully screened by icELISA using Tar-OVA as the coating antigen. A sensitive immunoassay was developed to characterize the sensitivity of monoclonal antibody 1F3. The standard inhibition curve was generated according to logistic curve fitting (four-parameter) and is shown in Figure 3. The monoclonal antibody 1F3 exhibited an IC_50_ value of 0.105 ng/mL, a limit of detection (LOD) of 0.014 ng/mL and a linear range of quantitation (LOQ) from 0.029 to 0.440 ng/mL for the tartrazine standard. Thirteen pigments with related structures were selected to characterize the specificity of mAb 1F3. The results, shown in Table 1, indicated that mAb 1F3 was highly specific for tartrazine and showed no cross-reactivities to other related pigments.

### 3.5. Fortification Experiment

The immunoassay we developed was employed to analyze orange juice in a fortification experiment. The orange juice was bought from a local supermarket and was previously proven to be a negative sample without any tartrazine additives. The fortified samples were measured by direct serial dilution without any pretreatment, due to the ultrasensitivity of the monoclonal antibody 1F3. The results are shown in Table 2. The obtained intra- and inter-assay recoveries for fortified orange juice ranged from 89.33% to 109.70% and from 81.33% to 93.43%, respectively. The intra-assay coefficients of variation were found to be from 7.54% to 12.35%, and the inter-assay values were between 5.90% and 10.48%. The established immunoassay was also performed to measure a real positive sample of carbonated beverage. The concentration of tartrazine measured from this carbonated beverage was calculated to be 13,456.7 ± 912.9 ng/mL based on the test of 18 replicates at three different dilution levels, and the coefficient of variation was found to be 6.78%, which indicated the immunoassay to be very reliable. The developed immunoassay was more sensitive than most of the instrumental analysis methods, and could be applied to the direct detection of tartrazine in soft drinks without any ultrasonication or complicated SPE cleanup pretreatments.

## 4. Conclusions

In this study, we successfully synthesized three haptens of tartrazine and their corresponding immunogens and coating antigens. The sulfonic acid groups on the hapten structure of tartrazine were thoroughly studied for the first time, and were found to have an enormous impact on the generation of antibodies specific to tartrazine. A most sensitive and specific monoclonal antibody against tartrazine was successfully produced, and the immunoassay developed was used to measure fortified samples of orange juice as well as real positive samples of a carbonated beverage with satisfactory results. The established icELISA method was very simple and ultrasensitive, and could be applied to the determination of tartrazine in food sample.

## Figures and Tables

**Table 1.** Cross reactivity of mAb 1F3 with tartrazine and other related pigments.

**Compound**	**Structure**	**IC_50_ (ng/mL)**	**CR(%)**
Erythrosine	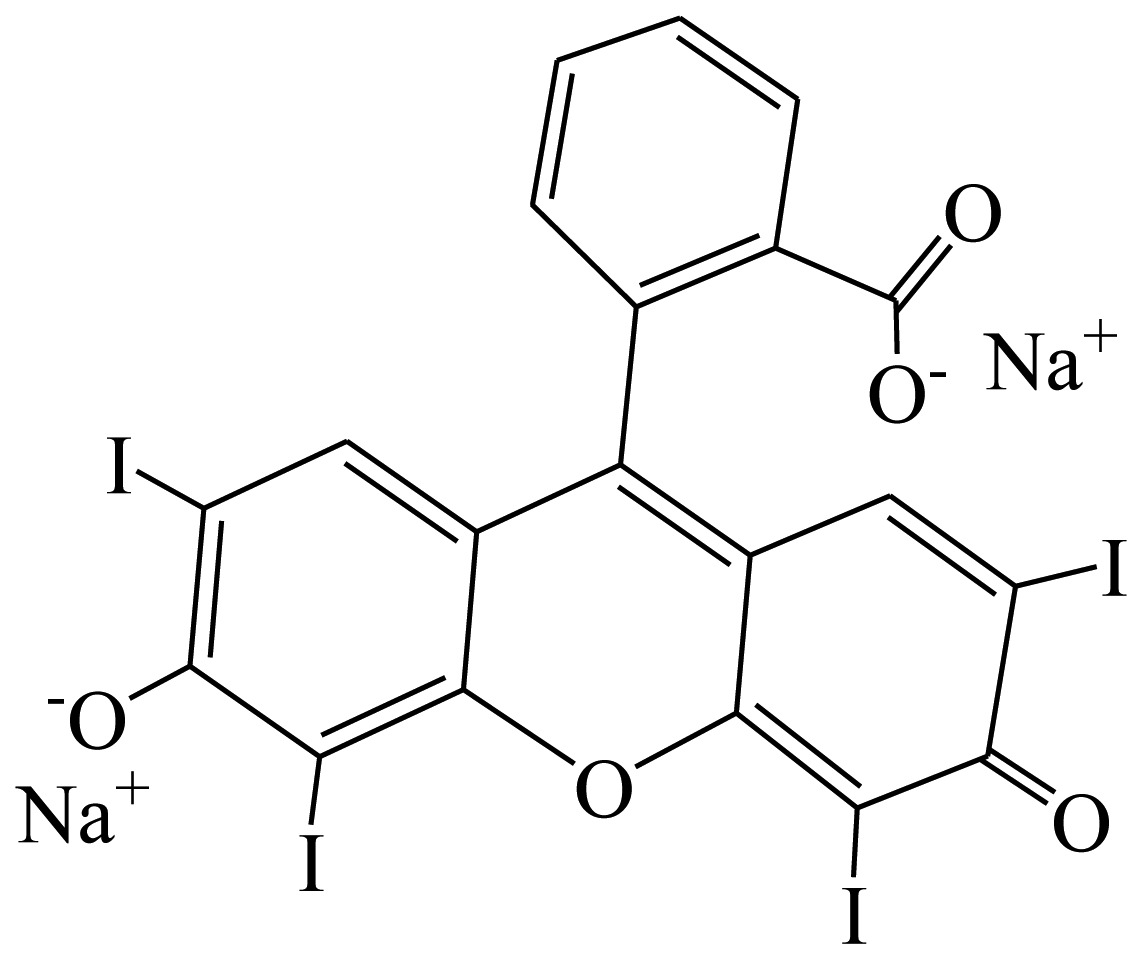	^a^ ND	<1
Crystal Violet	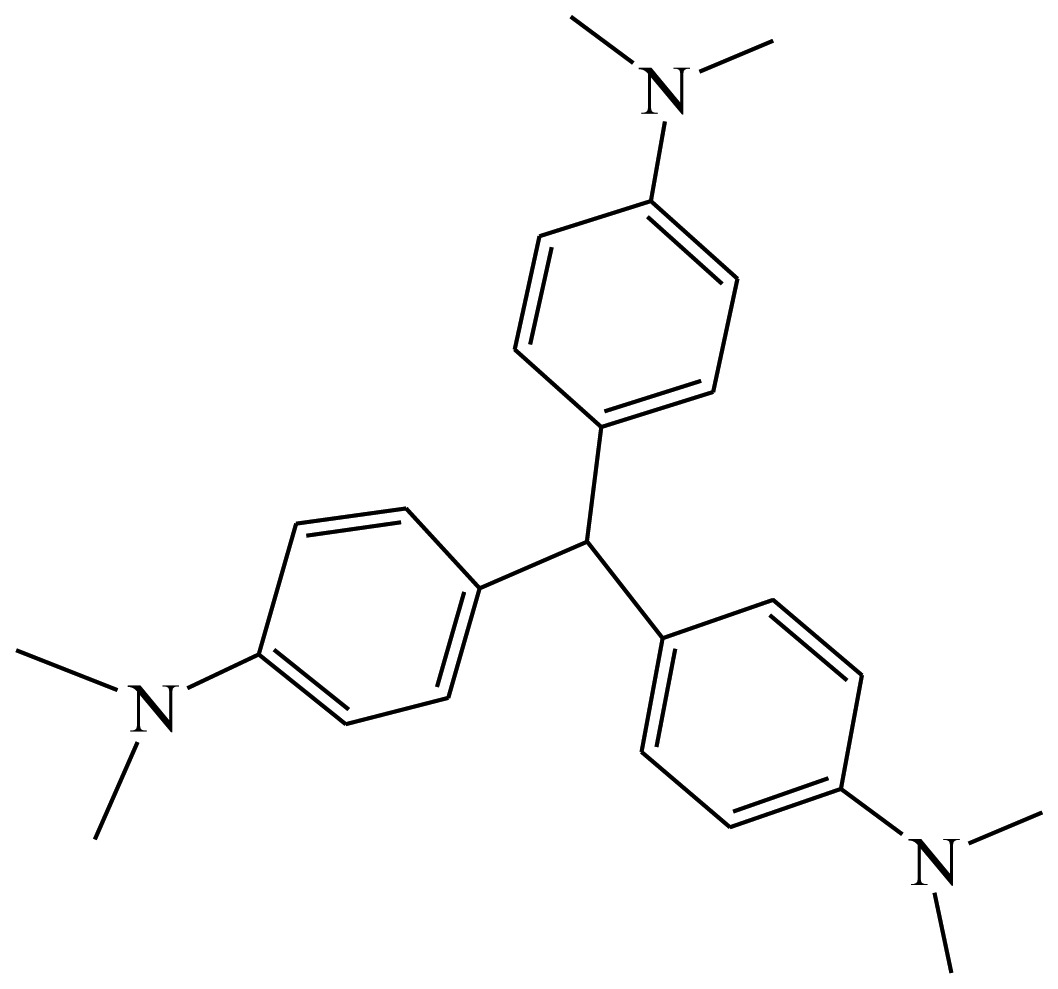	ND	<1
Malachite green	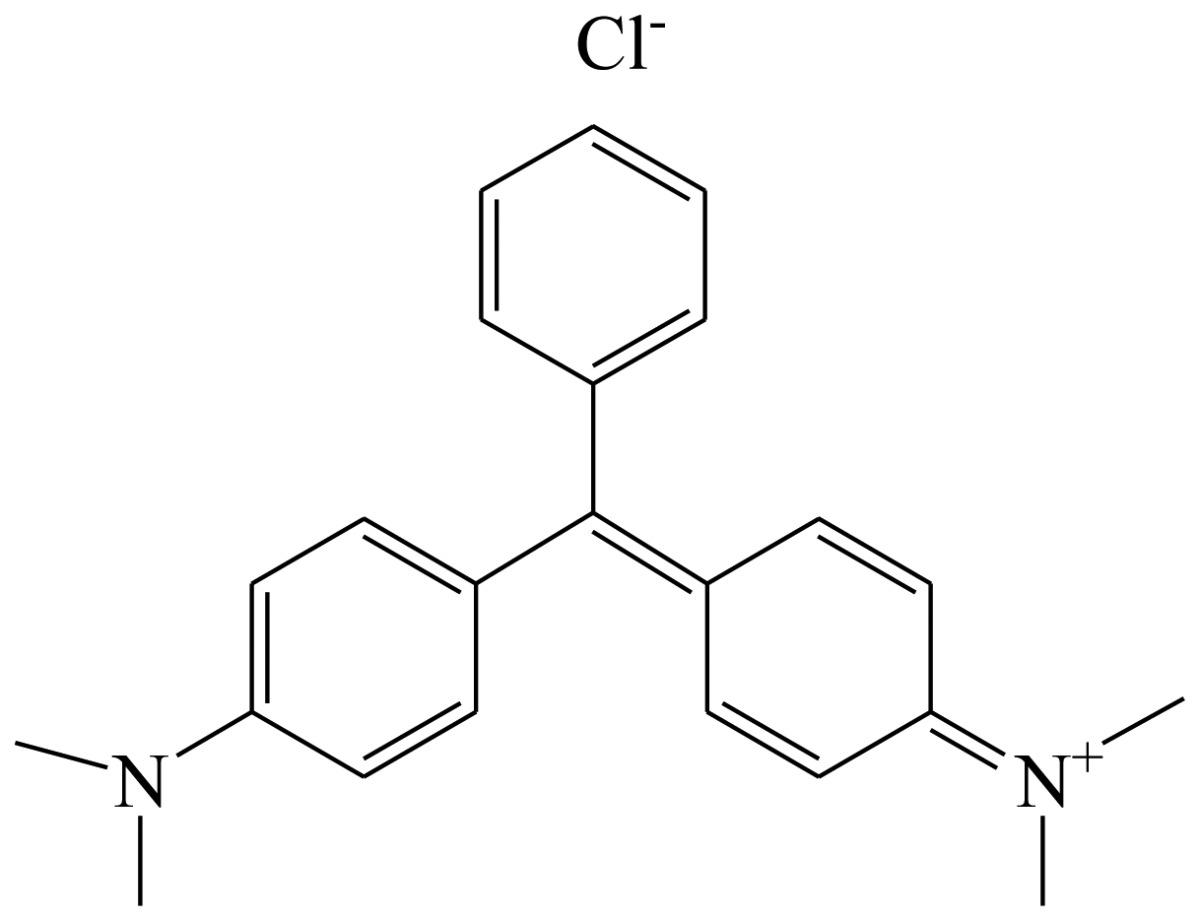	ND	<1
Tartrazine	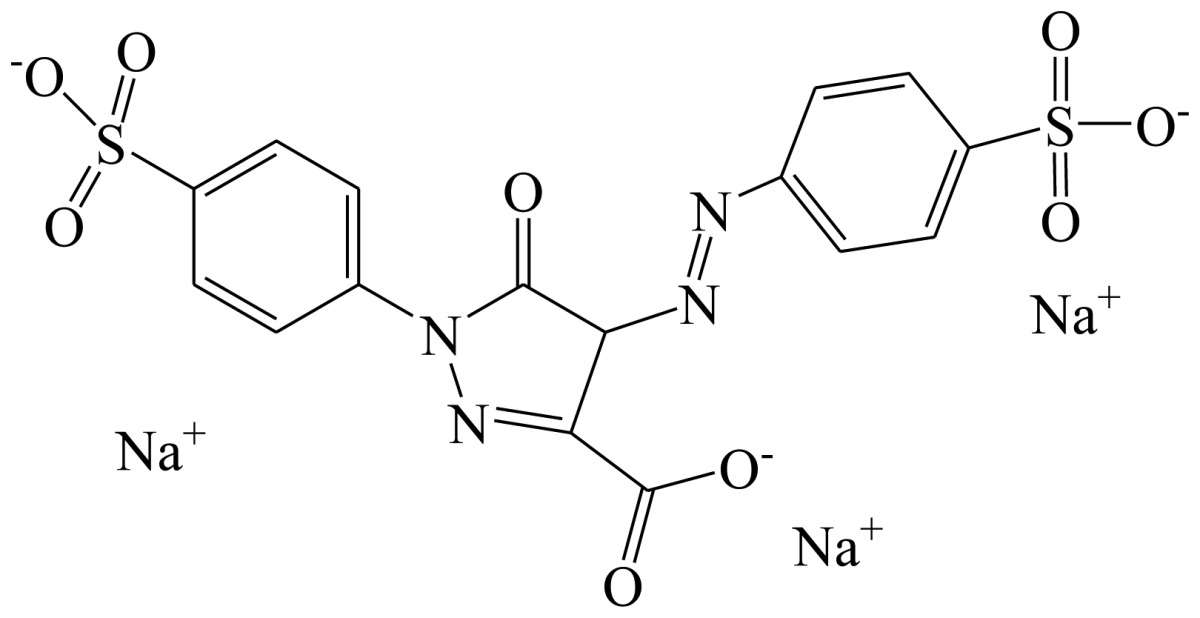	0.11	100%
Sunset Yellow	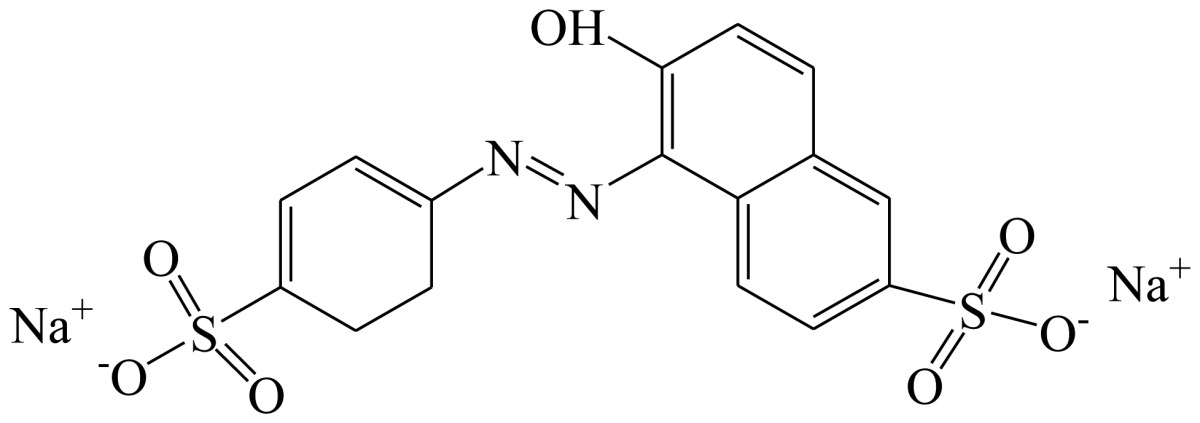	ND	<1
New coccine	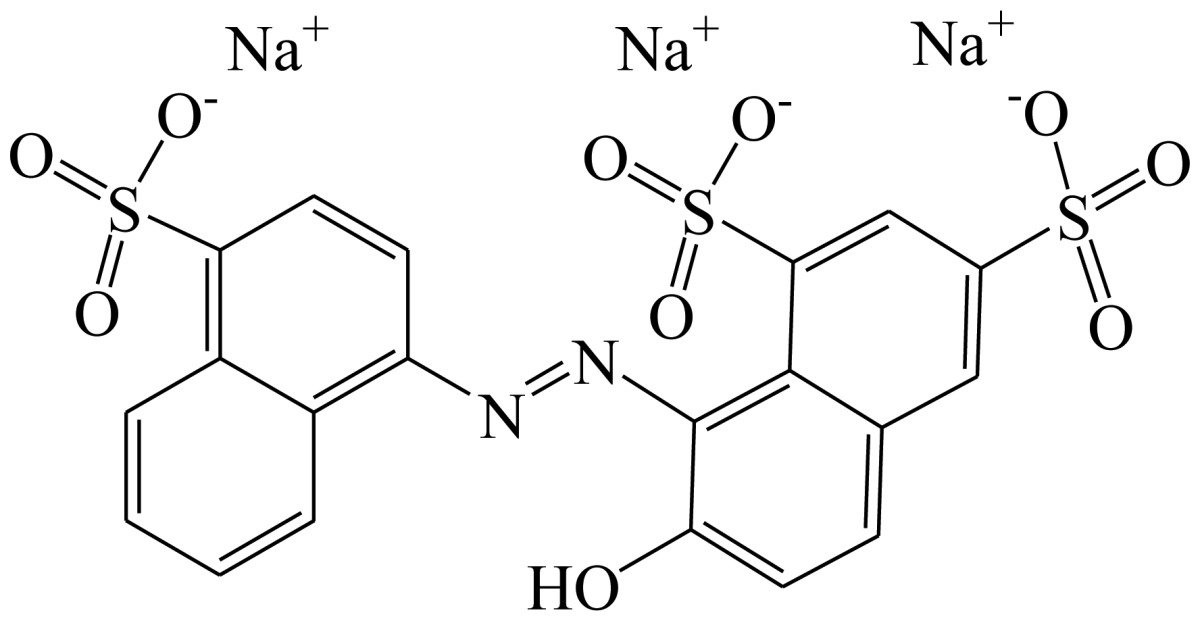	ND	<1
Allura Red AC	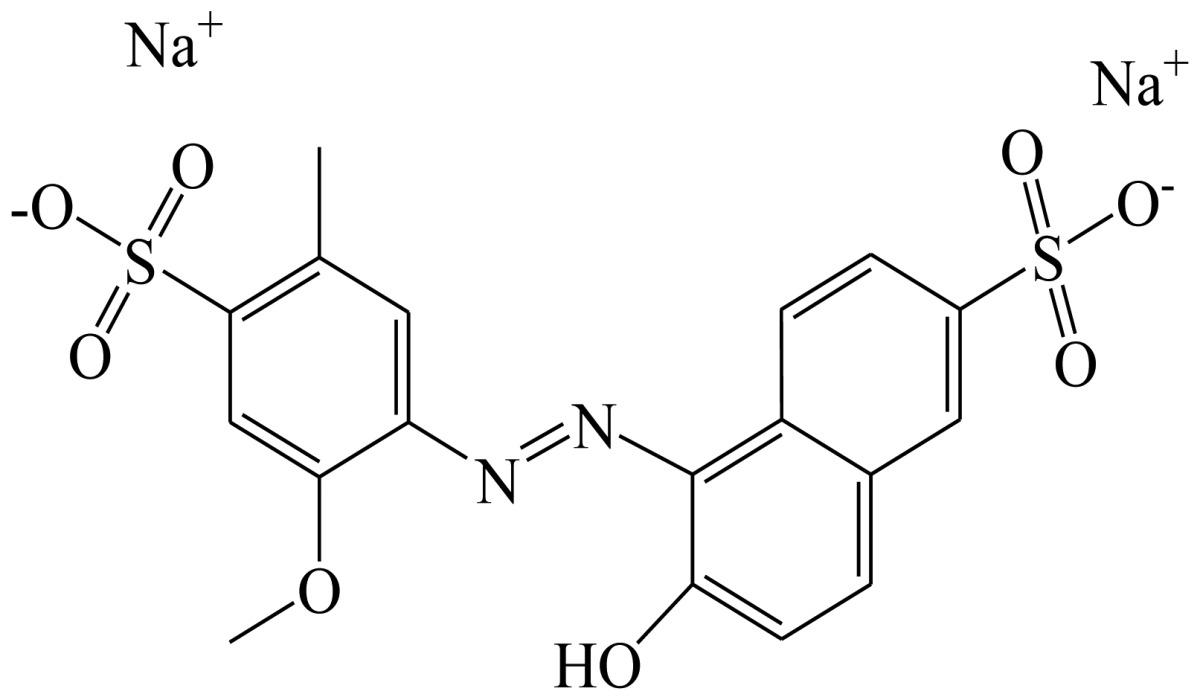	153.23	<1
Orangeim	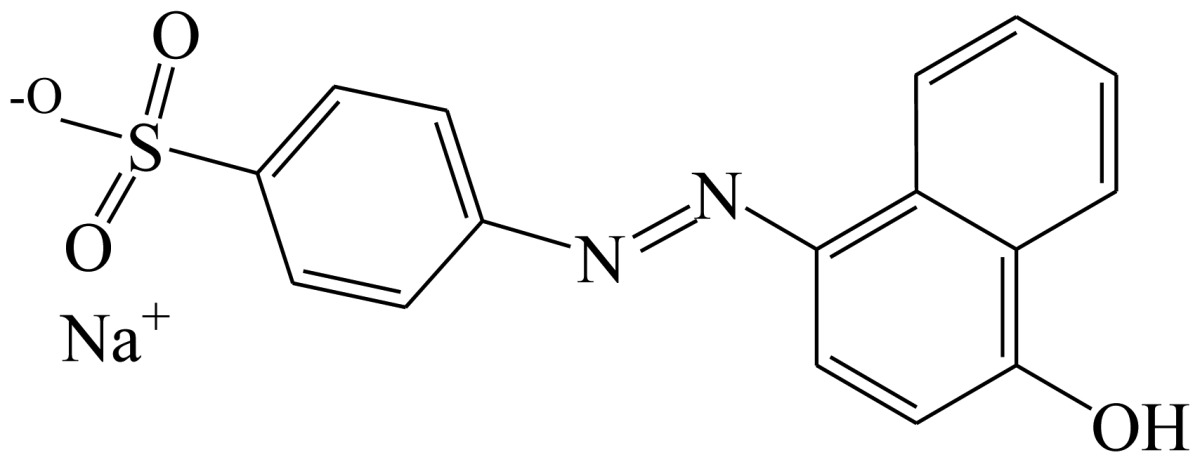	ND	<1
Auramine O	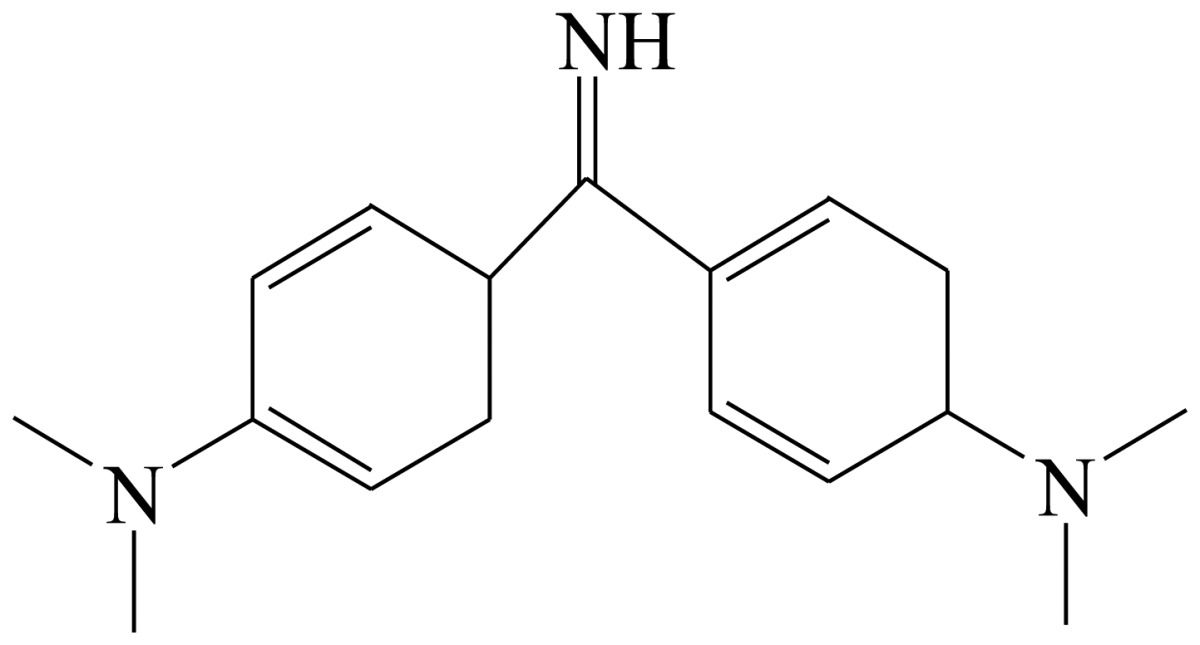	ND	<1
Sudan II	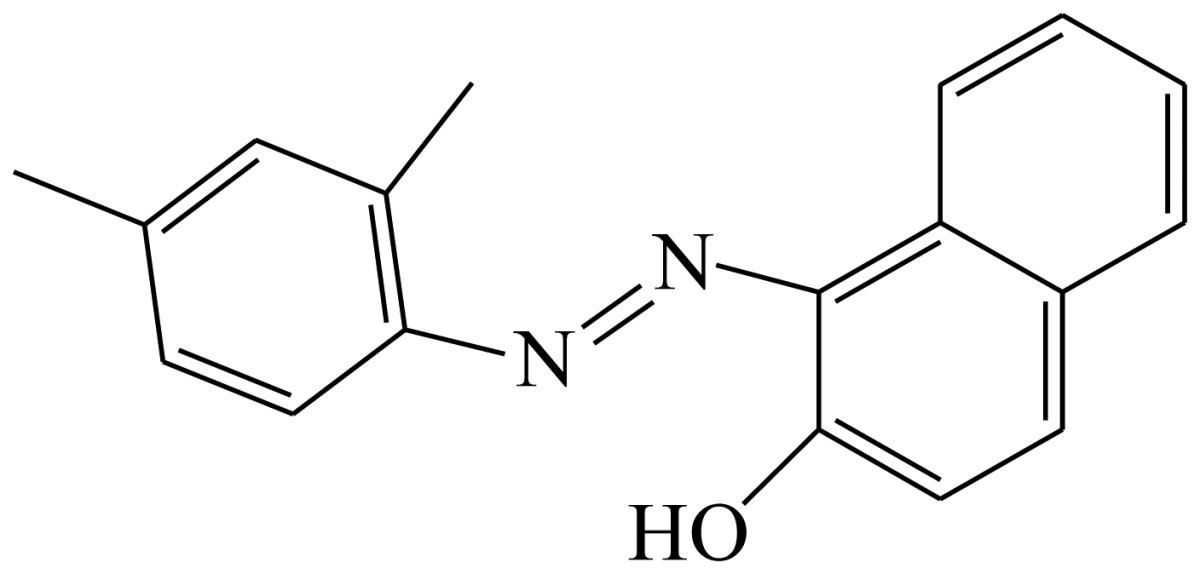	ND	<1
Solvent Red 24	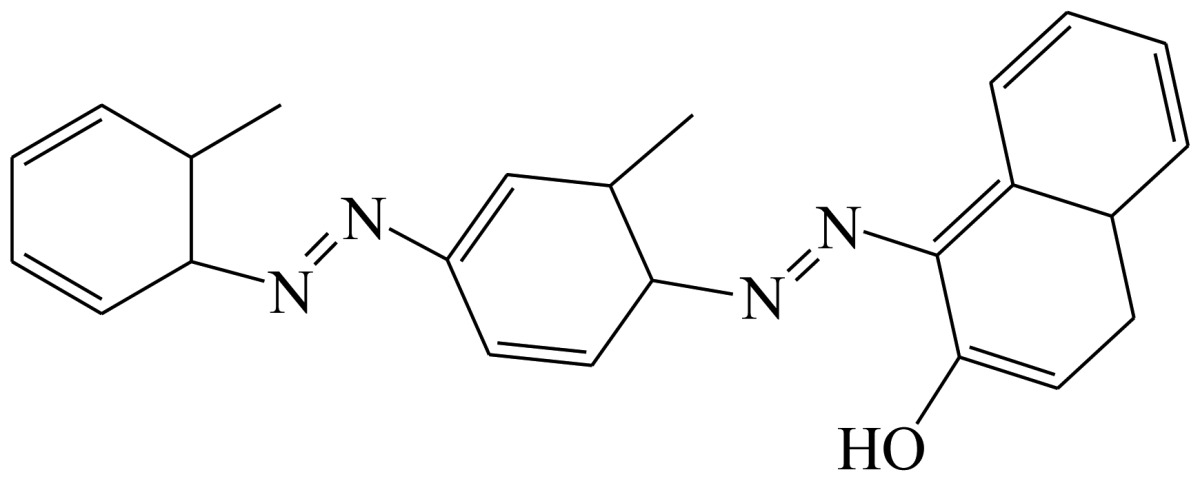	ND	<1
Solvent Red 1	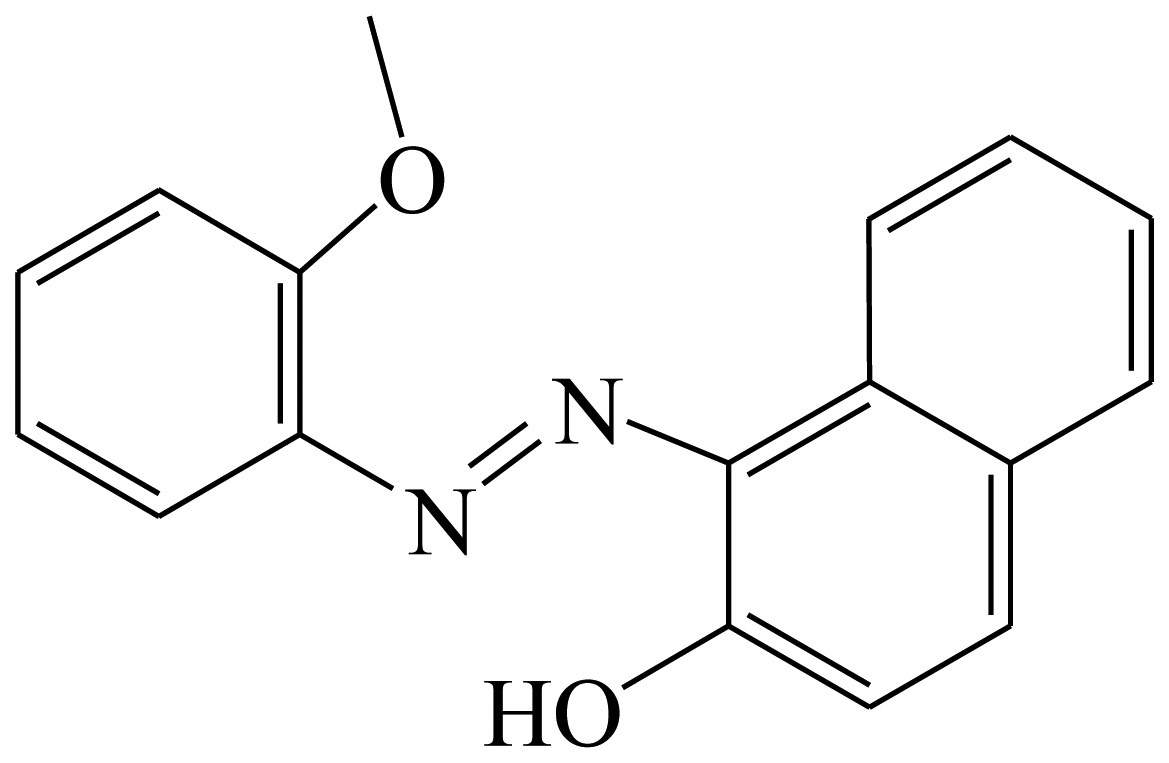	ND	<1
Indigotin	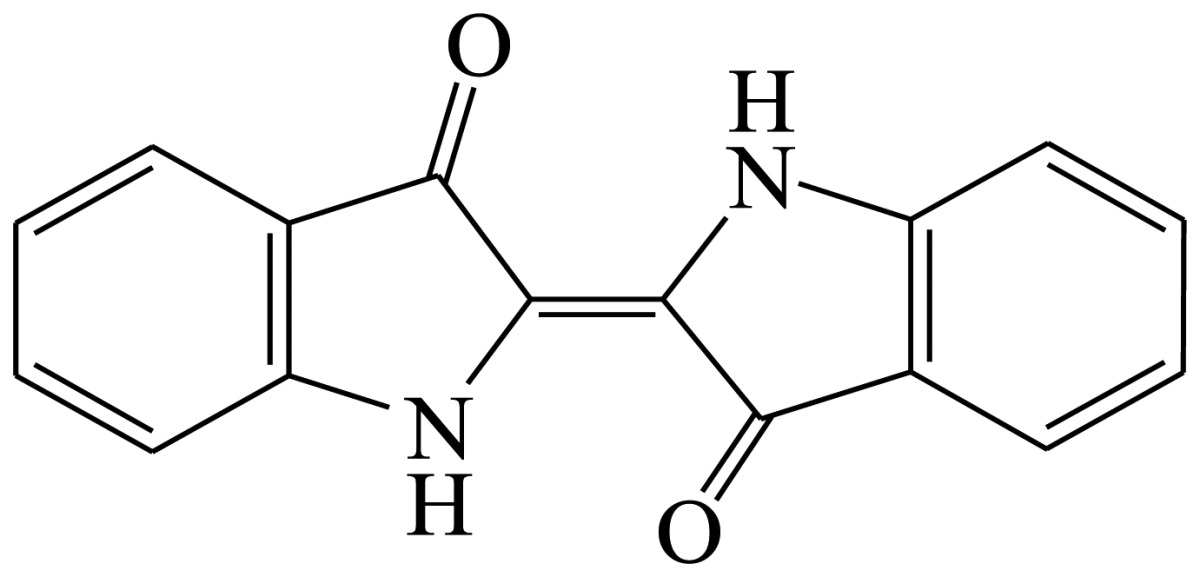	ND	<1
Chrysodine	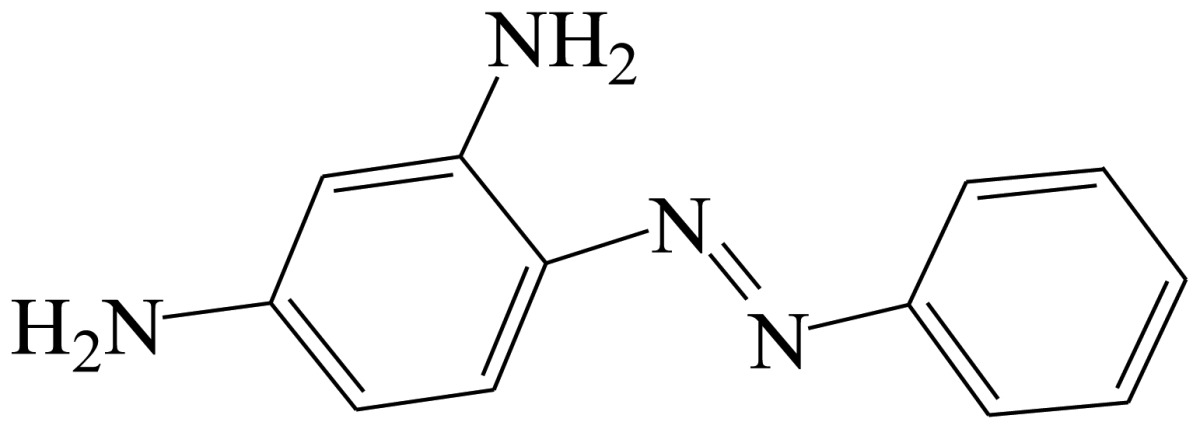	ND	<1

a ND represents the infinite IC_50_ could not be fitted with the four-parameter logistic equation.

**Table 2.** Recovery of tartrazine from fortified samples of orange juice by icELISA.

		**Intra-Assay (n = 5)**			**Inter-Assay (n = 5)**		
			
**Fortified Level (mg/mL)**	**Diluted Level (ng/mL)**	**Measure Level (ng/mL)**	**Recovery**	**Coefficient of Variation**	**Measure Level (ng/mL)**	**Recovery**	**Coefficient of Variation**
0.5	0.1	0.110 ± 0.008	109.70%	7.54%	0.093 ± 0.006	93.43%	5.90%
1	0.2	0.196 ± 0.022	98.17%	11.26%	0.163 ± 0.017	81.33%	10.48%
2	0.4	0.357 ± 0.044	89.33%	12.35%	0.336 ± 0.023	84.12%	6.70%
